# Genetics of Sputum Gene Expression in Chronic Obstructive Pulmonary Disease

**DOI:** 10.1371/journal.pone.0024395

**Published:** 2011-09-16

**Authors:** Weiliang Qiu, Michael H. Cho, John H. Riley, Wayne H. Anderson, Dave Singh, Per Bakke, Amund Gulsvik, Augusto A. Litonjua, David A. Lomas, James D. Crapo, Terri H. Beaty, Bartolome R. Celli, Stephen Rennard, Ruth Tal-Singer, Steven M. Fox, Edwin K. Silverman, Craig P. Hersh, and the ECLIPSE Investigators

**Affiliations:** 1 Channing Laboratory, Brigham and Women's Hospital, Harvard Medical School, Boston, Massachusetts, United States of America; 2 Division of Pulmonary and Critical Care Medicine, Brigham and Women's Hospital, Harvard Medical School, Boston, Massachusetts, United States of America; 3 GlaxoSmithKline, Uxbridge, United Kingdom; 4 GlaxoSmithKline, Research Triangle Park, North Carolina, United States of America; 5 Medicines Evaluation Unit, University of Manchester, Manchester, United Kingdom; 6 Department of Thoracic Medicine, Haukeland University Hospital, Institute of Medicine, University of Bergen, Bergen, Norway; 7 Cambridge Institute for Medical Research, University of Cambridge, Cambridge, United Kingdom; 8 Department of Medicine, National Jewish Health, Denver, Colorado, United States of America; 9 Department of Epidemiology, Bloomberg School of Public Health, Johns Hopkins University, Baltimore, Maryland, United States of America; 10 Division of Pulmonary and Critical Care Medicine, University of Nebraska Medical Center, Omaha, Nebraska, United States of America; 11 GlaxoSmithKline, King of Prussia, Pennsylvania, United States of America; University of Washington, United States of America

## Abstract

Previous expression quantitative trait loci (eQTL) studies have performed genetic association studies for gene expression, but most of these studies examined lymphoblastoid cell lines from non-diseased individuals. We examined the genetics of gene expression in a relevant disease tissue from chronic obstructive pulmonary disease (COPD) patients to identify functional effects of known susceptibility genes and to find novel disease genes. By combining gene expression profiling on induced sputum samples from 131 COPD cases from the ECLIPSE Study with genomewide single nucleotide polymorphism (SNP) data, we found 4315 significant cis-eQTL SNP-probe set associations (3309 unique SNPs). The 3309 SNPs were tested for association with COPD in a genomewide association study (GWAS) dataset, which included 2940 COPD cases and 1380 controls. Adjusting for 3309 tests (p<1.5e-5), the two SNPs which were significantly associated with COPD were located in two separate genes in a known COPD locus on chromosome 15: *CHRNA5* and *IREB2*. Detailed analysis of chromosome 15 demonstrated additional eQTLs for *IREB2* mapping to that gene. eQTL SNPs for *CHRNA5* mapped to multiple linkage disequilibrium (LD) bins. The eQTLs for *IREB2* and *CHRNA5* were not in LD. Seventy-four additional eQTL SNPs were associated with COPD at p<0.01. These were genotyped in two COPD populations, finding replicated associations with a SNP in *PSORS1C1*, in the *HLA-C* region on chromosome 6. Integrative analysis of GWAS and gene expression data from relevant tissue from diseased subjects has located potential functional variants in two known COPD genes and has identified a novel COPD susceptibility locus.

## Introduction

Gene expression levels in humans are highly heritable [1], [2]. Multiple published studies have examined the associations between single nucleotide polymorphism (SNP) variation and microarray gene expression measurements to identify expression Quantitative Trait Loci (eQTLs ), single nucleotide polymorphisms (SNPs) that influence gene expression [3]–[5]. However, most of the published studies have examined gene expression in lymphoblastoid cell lines (LCL) from unphenotyped individuals [3], [4], though a recent paper has described eQTLs in peripheral blood CD4+ lymphocytes of patients with asthma [6]. Integrative genomic analyses can provide functional information regarding significant SNPs found through genomewide association studies (GWAS) or identify the key genes within a locus identified through GWAS. For example, genome-wide expression profiling in LCL from children with asthma [5] was used to localize *ORMDL3* (ORM1-like 3 (S. cerevisiae) [MIM 610075]) as the likely gene for childhood asthma in the multi-gene chromosome 17q21 locus found through GWAS [7]. However, this study did not determine whether the eQTLs identified were relevant in primary human tissues in asthma. Integrative genomics studies can also be used to implicate novel genes for complex traits, such as the association between *MMP20* (matrix metallopeptidase 20 [MIM 604629]) and age related decline in kidney function [8].

Chronic obstructive pulmonary disease (COPD [MIM 606963]), which includes emphysema and chronic bronchitis, is a complex disease with genetic and environmental influences [9]. COPD is a major source of morbidity and mortality in the U.S. and worldwide [10]. Previous GWAS have identified three susceptibility loci for COPD, including *HHIP* (hedgehog interacting protein [MIM 606178]), *FAM13A* (family with sequence similarity 13, member A [MIM 613299]), and a multi-gene locus on chromosome 15q25 containing candidate genes *CHRNA5* (cholinergic receptor, nicotinic, alpha 5 [MIM 118505]), *CHRNA3* (MIM 118503), and *IREB2* (iron-responsive element binding protein 2 [MIM 147582]) [11]–[13]. Cough and phlegm production is common among COPD patients, and sputum samples may provide a non-invasive window into pathobiologic processes in the lungs of COPD patients. Therefore, we integrated GWAS data with microarray gene expression profiles from induced sputum samples from well-characterized COPD subjects participating in the Evaluation of COPD Longitudinally to Identify Predictive Surrogate End-points (ECLIPSE) Study [14]. We addressed two hypotheses: (1) eQTL analysis will improve understanding of previously known COPD susceptibility loci, such as chromosome 15q25; and (2) eQTL SNPs can be used to identify novel COPD susceptibility genes. Limiting the search to functional eQTL SNPs can reduce the multiple testing burden found in traditional GWAS. Although eQTL studies have now been performed in several human tissues besides blood, our study represents one of the first integrative genomics analyses performed in affected patients in order to gain insights into a common disease.

## Methods

### Ethics Statement

Study subjects provided written informed consent, and all studies were approved by the Institutional Review Boards at Partners Healthcare and all participating centers.

### ECLIPSE Study

ECLIPSE was a three year observational study conducted at 46 centers in 12 countries [14]. ECLIPSE recruited 2083 COPD subjects ages 40–75 with a smoking history of at least 10 pack-years (cigarettes smoked per day multiplied by years smoked, divided by 20 to convert to packs), 332 control smokers with at least 10 pack-years smoking history and normal lung function, and 237 non-smoking controls [15]. COPD was defined by GOLD stage 2 or greater (FEV_1_/FVC<0.7 with FEV_1_<80% predicted) [10]. Genome-wide SNP genotyping was performed on all ECLIPSE subjects using the Illumina HumanHap550 BeadChip. GWAS analysis included 1736 cases COPD cases and 175 controls [11]. Sputum induction was performed on a subset of COPD cases at 14 sites, using a standard protocol [16]. RNA was extracted from sputum cell pellets using TRIzol and amplified with the Nugen Ovation RNA Amplification kit. Gene expression profiling was performed on RNA extracted from sputum samples of 145 COPD cases (all ex-smokers) using the Affymetrix Human U133 Plus2 array [17]. MIAME-compliant array data are available in the Gene Expression Omnibus database (http://www.ncbi.nlm.nih.gov/geo), accession GSE22148. Only Caucasian subjects were included in this analysis.

### Other GWAS Populations

Subjects from two additional COPD case-control studies were merged with the ECLIPSE subjects in the combined GWAS analysis and the GWAS meta-analysis [11]. COPD cases and control smokers were Caucasians recruited in Bergen, Norway [18], [19]. Cases were defined by GOLD stage 2 or greater COPD; smoking controls had normal lung function. Both cases and controls had smoking history of at least 2.5 pack-years. GWAS included 838 cases and 791 controls, genotyped using the Illumina HumanHap550 BeadChip [12].

The National Emphysema Treatment Trial (NETT) cases have FEV_1_≤45% predicted and emphysema on chest CT scan [20], [21]. Thus, NETT cases have COPD severity of GOLD Stage 3 or greater. All NETT Genetics Ancillary Study subjects are former smokers; only white subjects are included in this analysis. The Normative Aging Study (NAS) is a cohort study of initially healthy men followed by the Boston VA [22]. To define a control group for comparison to NETT cases, we selected Caucasian subjects meeting the following criteria: FEV_1_>80% predicted, FEV_1_/FVC>90% predicted, at least 10 pack-years of smoking, and an adequate DNA sample [23]. Genomewide SNP genotyping has been performed in the NETT-NAS study (366 cases, 414 controls) using the Illumina 610-Quad BeadChip [11].

### Replication Populations

The International COPD Genetics Network (ICGN) was a family-based study of COPD at ten centers in North America and Europe [18], [24]. Probands were ages 45–65 with post-bronchodilator FEV_1_<60% predicted, FEV_1_/VC<90% predicted, a smoking history of at least 5 pack-years, and at least one sibling with ≥5 pack-year smoking history. Genotyping was performed on Caucasian subjects only (Table 1).

**Table 1:** Characteristics of Evaluation of COPD Longitudinally to Identify Predictive Surrogate End-points (ECLIPSE) study subjects in the integrative genomics analysis as well as subjects from the International COPD Genetics Network (ICGN) and the Genetic Epidemiology of COPD study (COPDGene) included in follow-up analyses.

	ECLIPSE	ICGN		COPDGene	
	Cases	Probands	Relatives	Cases	Controls
N	131	983	1876	496	498
Male sex	87 (66.4%)	580 (59.0%)	970 (51.7%)	244 (49.2%)	251 (50.4%)
Age	64.9 (±5.5)	58.4 (±5.4)	57.9 (±9.5)	64.7 (±8.1)	60.3 (±8.6)
Pack-years of smoking	46.8 (±28.3)	52.7 (±29.5)	39.2 (±25.3)	54.8 (±26.8)	38.8 (±21.0)
Current smoker	0	320 (32.6%)	956 (51.0%)	149 (30.0%)	168 (33.7%)
Post-bronchodilator FEV_1_, % predicted	49.3 (±15.3)	35.5 (±13.1)	83.3 (±26.0)	48.8 (±18.4)	98.0 (±11.3)
Post-bronchodilator FEV_1_/FVC	0.43 (±0.12)	0.37 (±0.12)	0.64 (±0.14)	0.48 (±0.13)	0.78 (±0.05)

Values are presented as mean (±SD) or N (%).

FEV_1_ = forced expiratory volume in 1 second.

FVC = forced vital capacity.

The Genetic Epidemiology of COPD Study (COPDGene) enrolled COPD cases and control smokers at 21 clinical centers throughout the United States [25]. Subjects are 45–80 years old and have a smoking history of at least 10 pack-years. This analysis included the first 994 non-Hispanic white case and control subjects enrolled in COPDGene (Table 1). In these samples, a set of 75 ancestry informative markers has been previously genotyped and did not show evidence of population stratification [11].

SNP genotyping in ICGN and COPDGene SNPs was done using the iPLEX Gold assay on the Sequenom (San Diego, CA) MassARRAY system [26] or the TaqMan 5′ exonuclease assay (Applied Biosystems, Foster City, CA) [27].

### Statistical Analysis

A total of 145 COPD subjects had sputum samples with gene expression data available; two arrays failed quality control. Of the remaining 143 subjects, 131 had corresponding genomewide SNP data and phenotype data. The Affymetrix HG-U133 Plus 2 array contains 54,675 probe sets. After filtering out 17,420 probe sets which were not annotated with a specific gene symbol in the hgu133plus2.db R/Bioconductor database or which mapped to the X or Y chromosomes, 37,255 probe sets remained. Microarray preprocessing used the robust multiarray average method and quantile normalization [28], implemented in Bioconductor. QC of microarrays was performed using the Bioconductor package affyQCReport; QC results are available in the Data S1 and in Figure S1. QC of genomewide SNP data in ECLIPSE has been reported [11]. SNPs with minor allele frequency <0.05 in the 131 ECLIPSE cases were additionally excluded.

In the integrative analysis, each expression probe set was mapped to its corresponding gene and all genotyped SNPs were identified within 50 kb of the transcription start site (TSS). General linear models were used to detect cis-acting associations between probe set expression levels and SNP genotypes, adjusted for age, gender, and the first six genetic ancestry principal components derived from the genotype data on all ECLIPSE COPD cases [29]. False discovery rate adjusted p-value<0.05 defined statistical significance. eQTL analysis utilized the GGTools Bioconductor package [30].

Each significant cis-eQTL SNP was then tested for association with COPD in the combined GWAS dataset from ECLIPSE, Norway, and NETT-NAS [11]. The published combined GWAS analysis was a mega-analysis of individual-level genotype data, using logistic regression, adjusted for age, pack-years of smoking and principal components for genetic ancestry. In the published meta-analysis, stratified logistic regression was performed within each case-control study and results were combined using Z-scores for weighting by the inverse variance. SNPs associated with COPD at p<0.01 in either the combined GWAS analysis (mega-analysis) or the GWAS meta-analysis were genotyped for replication in ICGN and COPDGene. In the COPDGene study, case-control data were analyzed with linear regression models, adjusted for age, sex, and pack-years of smoking, using PLINK version 1.0.7 [31]. Family-based ICGN data were analyzed in PBAT version 3.6.1, adjusted for age, sex, and pack-years of smoking [32].

We also tested for eQTL SNPs influencing the expression of genes in previously identified COPD loci. On chromosome 15q25, we defined a region starting 50 kb centromeric from *IREB2* extending 50 kb telomeric from *CHRNB4* (approx. 300 kb total) and tested all genotyped SNPs within this region for association with expression levels of probe sets for six genes: *IREB2*, *AGPHD1*, *PSMA4*, *CHRNA5*, *CHRNA3*, and *CHRNB4*. For the other two COPD loci, we expanded the cis-eQTL analysis to all SNPs with 200 kb of the TSS of the genes *HHIP* and *FAM13A*.

## Results

### Sputum eQTL Analysis

**Figure 1:**
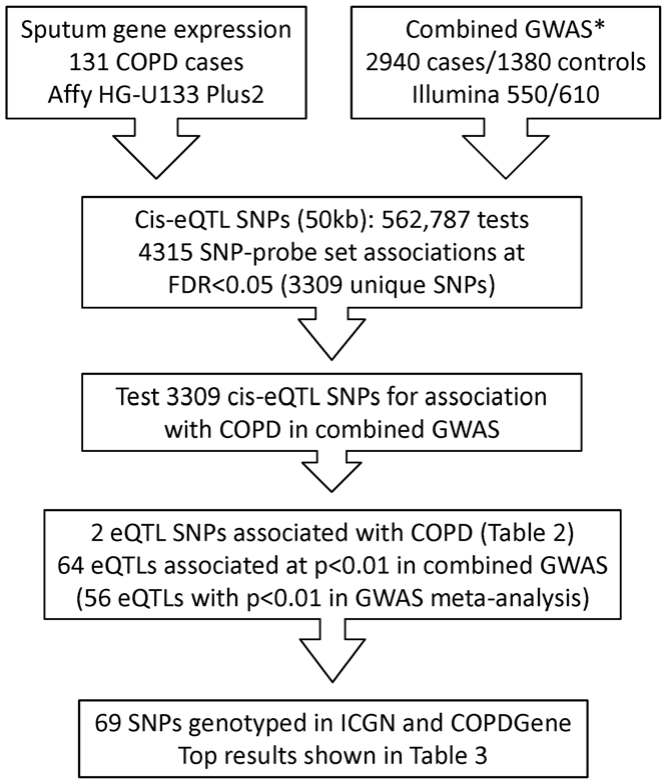
Overview of integrative genomics data analysis. *Combined genomewide association study (GWAS) = Evaluation of COPD Longitudinally to Identify Predictive Surrogate End-points (ECLIPSE), Bergen Norway, and National Emphysema Treatment Trial (NETT)-Normative Aging Study (NAS) [11]. COPD = chronic obstructive pulmonary disease. eQTL = expression quantitative trait locus. FDR = false discovery rate. ICGN = International COPD Genetics Network. SNP = single nucleotide polymorphism.

Characteristics of the 131 ECLIPSE COPD subjects in the eQTL analysis are shown in Table 1. On average, COPD subjects had a heavy smoking history and severely impaired lung function, similar to the full set of ECLIPSE GWAS cases [11]. The data analysis is outlined in Figure 1. Combining the gene expression data with genomewide SNP data and limiting analysis to potential cis-acting SNPs (within 50 kb of TSS) yielded 562,787 SNP-probe set association tests. Of these, 4315 SNP-probe set associations were significant at FDR-adjusted p<0.05 (corresponding to unadjusted p = 3.8e-4), representing 3309 unique SNPs and 1399 unique probe sets, covering 1086 genes (Table S1).

The top eQTL was for SNP rs104664 within the gene *FAM118A* (family with sequence similarity 118, member A). This SNP was found to be highly associated with *FAM118A* expression (Affymetrix Human 1.0 ST Exon array) in human osteoblasts [33], suggesting cross-tissue generalizability of this eQTL association. Other significant eQTL associations observed in sputum included *CHURC1* (churchill domain containing 1 [MIM 608577]), *HLA-DQB1* (major histocompatibility complex, class II, DQ beta 1 [MIM 604305]) and *HLA-DQA1* (MIM 146880), all of which were previously been found in other tissues, such as LCL [4], [34] and brain [35], according to a search of the GTEx (Genotype-Tissue Expression) eQTL Browser (http://www.ncbi.nlm.nih.gov/gtex, accessed 11/30/2010).

### Sputum eQTLs associated with COPD

We queried the 3309 significant cis-eQTL SNPs in the combined GWAS dataset including ECLIPSE, Norway, and NETT-NAS subjects [11]. Using a strict Bonferroni correction, there were two cis-eQTL SNPs significantly associated with COPD at p<0.05/3309 = 1.5e-5 (Table 2). These two SNPs on chromosome 15q25 are located in *CHRNA5* and *IREB2*, genes with known COPD associations.[12], [36] At a nominal threshold of p<0.01, there were 64 cis-eQTL SNPs associated with COPD (Table S2). There were 56 eQTL SNPs associated with COPD at p<0.01 in the meta-analysis of the ECLIPSE, Norway, NETT-NAS GWAS studies (Table S3), as opposed to the combined analysis of individual-level genotype data. Merging the 64 SNPs from the combined GWAS analysis and the 56 SNPs from the GWAS meta-analysis left 76 unique SNPs, which were brought to replication analysis.

**Table 2:** Sputum cis-eQTL SNPs significantly associated with COPD in the combined COPD GWAS.

Chrom	location	SNP	Affy probe	Gene	eQTL pvalue	FDR-adjusted pvalue	GWAS COPD OR^a^	GWAS COPD pvalue^a^	GWAS pvalue (adjusted)^b^
15	76532762	rs2656069	1555476_at	*IREB2*	0.00030	0.042	0.75	6.8E-06	0.023
15	76681394	rs1051730	206533_at	*CHRNA5*	0.00015	0.026	1.29	2.8E-06	0.0093

a Combined COPD genomewide association study (GWAS) according to Cho et al. [11].

b Bonferroni correction for 3309 sputum expression quantitative trait locus (eQTL) single nucleotide polymorphisms (SNPs) tested.

### Replication Studies

**Figure 2:**
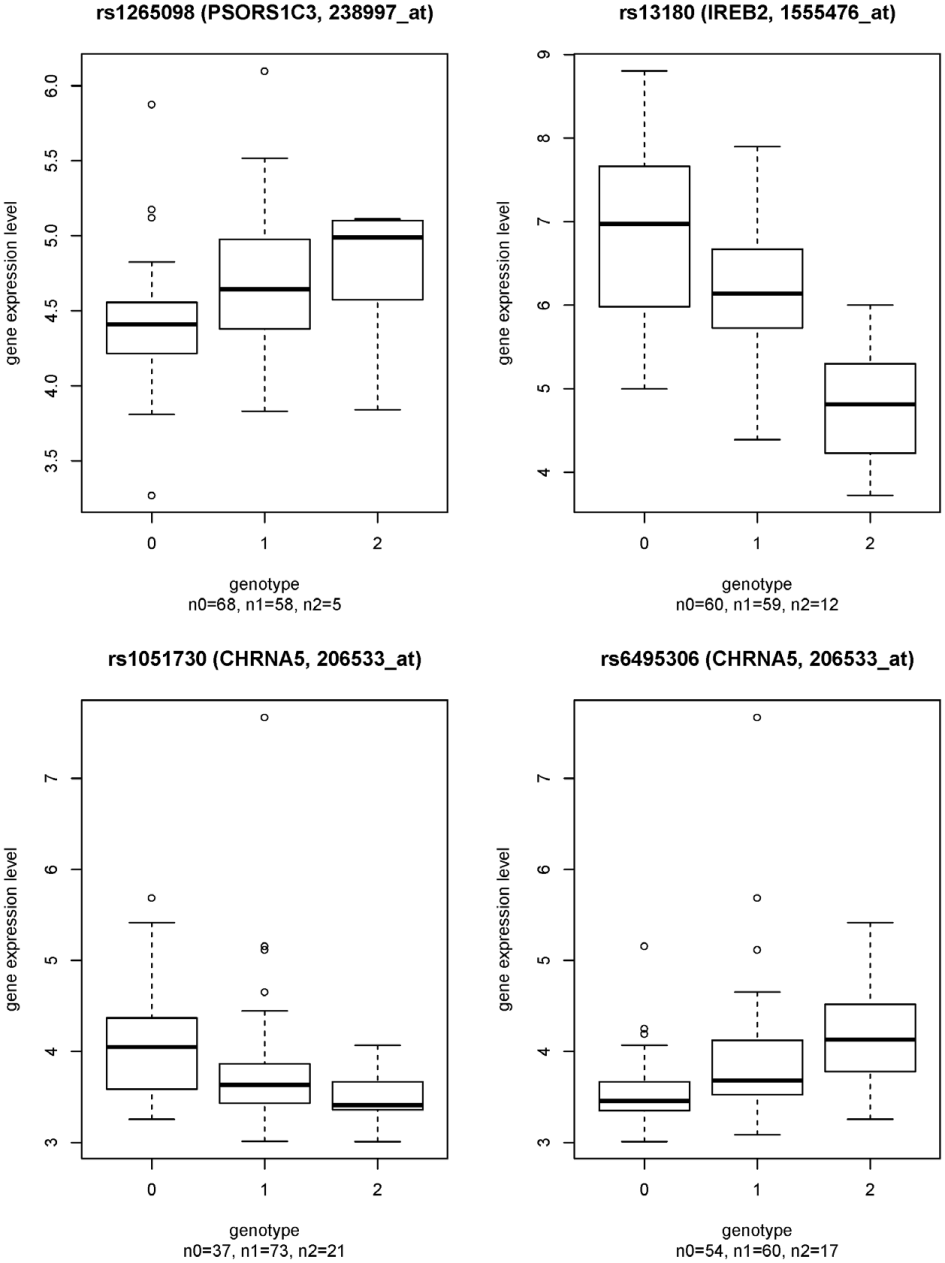
Boxplots of sputum gene expression levels stratified by genotype in 131 Evaluation of COPD Longitudinally to Identify Predictive Surrogate End-points (ECLIPSE) subjects with chronic obstructive pulmonary disease. a) rs1265098 - *PSORS1C3* (238997_at), p = 8.2e-5. b) rs13180 - *IREB2* (1555476_at), p = 6.7e-9. c) rs1051730 - *CHRNA5* (206533_at), p = 2.2e-4; LD bin 1 (see Table 4). d) rs6495306 - *CHRNA5* (206533_at), p = 9.9e-6; LD bin 3 (see Table 4).

Characteristics of the ICGN and COPDGene subjects in the replication analysis are reported in Table 1. The two SNPs in Table 2 were analyzed in previous reports [12], [36] and were not retested. Of the remaining 74 SNPs, 69 were successfully genotyped in ICGN and COPDGene. Screening in the larger ICGN study found 8 SNPs with p<0.1 (Table 3). Of these, only one had p<0.1 in COPDGene. SNP rs1265098 was significantly associated with COPD in ICGN and had a trend for significance in COPDGene. The effect direction for rs1265098 was consistent in ICGN, COPDGene, and the combined GWAS; the minor allele was associated with increased COPD risk in all three studies. SNP rs1265098 maps to the gene *PSORS1C1* (psoriasis susceptibility 1 candidate 1 [MIM 613525]) on chromosome 6, yet is associated with transcript levels of the neighboring gene *PSORS1C3* (p = 8.2e-05, FDR-adjusted p = 0.016) (Figure 2).

**Table 3:** Genetic association analysis of sputum expression quantitative trait locus (eQTL) single nucleotide polymorphisms (SNPs) with COPD susceptibility.

SNP	Chrom	BP	Gene	Effect on COPD risk in ICGN	ICGN pvalue	COPDGene OR	COPDGene pvalue	GWAS OR^a^	GWAS pvalue^a^
rs1999261	6	6515106	INTERGENIC	Increase	0.042	0.94	0.53	1.15	0.0080
rs1265098	6	31214156	*PSORS1C1*	Increase	0.024	1.20	0.098	1.18	0.0065
rs4750277	10	12954527	INTERGENIC	Increase	0.0062	0.97	0.80	0.83	0.0097
rs1025607	12	94884637	*AMDHD1*	Increase	0.011	1.12	0.26	a	a
rs2347279	18	2528545	*METTL4*	Decrease	0.097	1.01	0.91	0.83	0.0025
rs1878553	18	2560155	*NDC80*	Decrease	0.030	1.03	0.83	0.83	0.0028
rs4803481	19	46758396	INTERGENIC	Decrease	0.055	0.97	0.81	a	a
rs2302188	19	46777713	*CEACAM21*	Decrease	0.090	0.97	0.77	0.85	0.0075

SNPs with p-value<0.1 in the International COPD Genetics Network (ICGN) are shown.

a Odds Ratio and p-value from combined genomewide association study (GWAS) analysis [11] are shown, except as noted:

rs1025607 p = 0.0089 in GWAS meta-analysis.

rs4803481 p = 0.0043 in GWAS meta-analysis.

### Sputum eQTLs in COPD Candidate Loci

**Figure 3:**
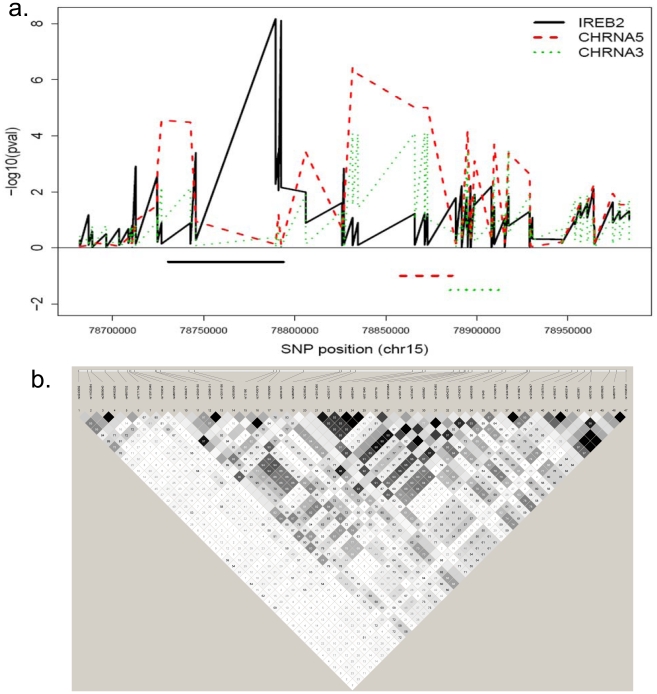
Detailed analysis of the chromosome 15q25 chronic obstructive pulmonary disease (COPD) locus. a) Association between single nucleotide polymorphisms (SNPs) in the chromosome 15q25 COPD locus and expression levels of *IREB2* (1555476_at), *CHRNA5* (206533_at) and *CHRNA3* (211587_x_at) in sputum samples from 131 Evaluation of COPD Longitudinally to Identify Predictive Surrogate End-points (ECLIPSE) subjects. SNP rs numbers are listed in Table 4. b) Linkage disequilibrium r^2^ values between SNPs in the chromosome 15q25 COPD locus (listed in Table 4) in 131 ECLIPSE subjects.

Previous GWAS have identified three loci associated with COPD susceptibility: *HHIP* on chromosome 4q31 [12], [13], *FAM13A* on chromosome 4q22 [11], and a region on chromosome 15q25 encompassing candidate genes *CHRNA5*, *CHRNA3* and *IREB2*, among others [12], [36]. On chromosome 15q25, cis-eQTL associations for *IREB2* mapped to that gene (Figure 3a). Genetic regulation of *CHRNA5* was more complex. Previous studies have demonstrated cis-acting effects of multiple SNPs on *CHRNA5* expression. Saccone et al. defined 4 LD bins surrounding *CHRNA5* with varying associations with cigarette smoking, lung cancer, and COPD [37]. Bins 1–3 were represented in our dataset, tagged by SNPs rs1051730, rs938682, and rs6495306, respectively (Table 4). SNPs in bins 1 and 3 were associated with *CHRNA5* expression in sputum (Figure 2), as has been demonstrated in brain [38] and lung tissue [39]. SNPs in bin 2 were not eQTLs for *CHRNA5*. We added additional SNPs to these bins, based on strong LD with tag SNPs in the larger ECLIPSE GWAS dataset. We also identified 3 sets of SNPs (3a, b, c in Table 4) with cis-eQTL associations for *CHRNA5* and moderate LD with SNPs in bin 3 (r^2^ 0.57–0.76). SNPs in bins 1 and 2, but not bin 3, showed evidence of association with COPD in the combined GWAS dataset, though they were not genomewide significant (bin 1: rs1051730, p = 2.8e-6; bin 2: rs938682, p = 5.6e-5; bin 3: rs6495306, p = 0.2). These results suggest that the COPD-associated SNP rs1051730 (bin 1) may influence phenotype by its effect on gene expression, while COPD-associated SNPs in bin 2 (tagged by rs938682) may exert their effect through other mechanisms. SNPs in bin 3, although eQTLs, were not associated with COPD risk.

**Table 4:** Single nucleotide polymorphism (SNP) associations with expression of *IREB2* (1555476_at) and *CHRNA5* (206533_at) in induced sputum samples from COPD subjects.

SNP	Position	*IREB2* eQTL pvalue	*CHRNA5* eQTL pvalue	LD bin	r^2^ with locus tag	r^2^ with rs13180
rs4243082	78682076	0.88	0.89			
rs11635084	78686823	0.066	0.82			
rs2869040	78688834	0.26	0.68			
rs6495296	78696492	0.32	0.71			
rs4887052	78703631	0.26	0.88			
rs7171749	78708715	0.41	0.44			
rs12913946	78709937	0.30	0.72			
rs7183034	78710766	0.16	0.66			
rs4887053	78712699	0.0012	0.11			
rs1394371	78724469	0.0028	0.031			
rs12903150	78724645	0.014	0.0014			
rs12899131	78726885	0.71	2.8E-05	*3a*	*0.57*	<0.01
rs10519198	78742754	0.73	3.3E-05	*3a*	*0.57*	<0.01
rs2656069	78745707	4.0E-04	0.11	*IREB2*		0.44
**rs13180**	78789488	6.7E-09	0.74	*IREB2*		
rs3743079	78791061	3.9E-04	0.063	*IREB2*		0.36
rs1062980	78792527	7.7E-09	0.78	*IREB2*		0.99
rs8034191	78806023	0.010	3.8E-04	1	0.90	
rs3885951	78825917	0.025	0.20			
rs2036534	78826948	0.0015	0.043	*2*	*0.79*	
rs12915366	78831753	0.83	3.7E-07	*3*	*0.80*	
rs2292117	78834689	0.80	6.4E-07	*3*	*0.82*	
**rs6495306**	78865893	0.79	9.9E-06	3		<0.01
rs680244	78871288	0.79	9.9E-06	3	0.99	
rs621849	78872861	0.79	9.9E-06	3	0.99	
rs578776	78888400	0.016	0.70	2	0.72	
rs12910984	78891627	0.0061	0.45	*2*	0.99	
**rs1051730**	78894339	0.016	2.2E-04	1		0.19
rs3743077	78894896	0.93	6.7E-05	*3*	*0.98*	
**rs938682**	78896547	0.0061	0.45	2		0.26
rs12914385	78898723	0.014	8.0E-04	*1*	*0.81*	
rs8042374	78908032	0.0064	0.53	*2*	*0.98*	
rs3743075	78909452	0.76	2.0E-04	*3b*	*0.76*	0.01
rs6495309	78915245	0.088	0.64	2	0.90	
rs1948	78917399	0.16	4.2E-04	*3c*	*0.60*	0.03
rs11636753	78928946	0.91	0.0023			
rs12441998	78929372	0.14	0.85			
rs1316971	78930510	0.083	0.89			
rs12594247	78946633	0.63	0.62			
rs17487514	78953785	0.10	0.068			
rs1996371	78956806	0.025	0.025			
rs6495314	78960529	0.025	0.025			
rs922691	78963994	0.14	0.0060			
rs8032156	78964498	0.51	0.66			
rs8038920	78974545	0.059	0.011			
rs4887077	78978364	0.049	0.029			
rs11638372	78983559	0.049	0.029			

Linkage disequilibrium (LD) bins for *CHRNA5* associations are defined as in Saccone et al. [37]. SNPs tags for LD bins are shown in bold. Additional SNPs added to bins based on LD in ECLIPSE genomewide association study dataset are shown in italics.

SNPs in *IREB2* were both cis-eQTLs for that gene (Table 4, Figure 2) and were associated with COPD in the combined GWAS (rs13180, p = 5.0e-7). Even though some of the significant eQTL SNPs for *CHRNA5* mapped to *IREB2* (bin 3a), SNPs in all 3 bins were not in LD with the *IREB2* eQTL SNPs (Figure 3b). No SNPs were significantly associated with *AGPHD1*, *PSMA4*, or *CHRNB4* gene expression. For the other two COPD GWAS loci, *HHIP* and *FAM13A*, we found no significant cis-eQTL SNPs within 50 kb, so we expanded the assessment of cis-eQTLs to all SNPs within 200 kb of the TSS of each gene. There were no significant cis-eQTLs within 200 kb of either *HHIP* or *FAM13A*.

## Discussion

In a cohort of well-characterized COPD subjects, we integrated genomewide SNP and gene expression data derived from induced sputum, a biologically-relevant tissue in COPD, to identify a set of eQTL SNPs affecting gene expression levels. The SNPs were then tested for association with the clinical phenotype of COPD; gene expression was not tested for association with disease status in this set of COPD cases only. Using the eQTL results, we implicated two distinct COPD susceptibility genes in a previously identified region of chromosome 15q25. Additionally, we provide evidence for a potential novel COPD susceptibility locus in the HLA region on chromosome 6.

The initial GWAS in COPD found significant associations on chromosome 15q25, with SNPs in the genes *CHRNA3* and *CHRNA5*, encoding two subunits of the nicotinic acetylcholine receptor [12]. This region has also been associated with lung cancer, peripheral arterial disease, and smoking behavior [37], [40]–[43], so it is not clear whether these genes have a direct effect on COPD susceptibility, or their effects are at least partially influenced through cigarette smoking, the major environmental risk factor for COPD [44], [45]. In terms of genetic regulation of expression of the chromosome 15q25 genes, we found similar eQTL associations with *CHRNA5* expression in induced sputum as has been found in brain [38] and lung tissue [39]. We found additional sputum eQTL SNPs for *CHRNA5* in moderate LD with previously defined eQTLs. The previous papers on brain and lung tissue gene expression did not report testing *IREB2*, a gene previously associated with COPD [11], [36]. The specific *IREB2* SNPs associated in GWAS (rs13180) [11] and in a candidate gene analysis of differentially expressed genes (rs2656069) [36] were in only moderate LD (r^2^ = 0.44) with each other, implying independent effects on *IREB2* expression. The *IREB2* and *CHRNA5* eQTL SNPs were not in LD with each other, suggesting the presence of at least two COPD susceptibility genes on chromosome 15q25. Previous studies have similarly used eQTL analyses to add functional information about genes identified through GWAS, including studies of asthma [7], celiac disease [46], and Crohn's disease [47]. However, these prior studies have examined gene expression in blood cells, and not primary disease tissues.

However, we did not finding significant cis-eQTL SNPs for two other known COPD loci, *HHIP* and *FAM13A*. The associated SNPs found through GWAS may exert their effects on phenotype via other mechanisms besides influencing gene expression. Alternatively, the GWAS SNPs may actually be eQTLs acting in other tissues besides sputum, such as alveolar or bronchial epithelial cells, which were not assessed in our study.

Besides improving understanding of the COPD susceptibility locus on chromosome 15q25, we identified a potential novel COPD locus on chromosome 6. The SNP maps to gene *PSORS1C1*, but it is associated with expression levels of the neighboring gene *PSORS1C3*. Variants in *PSORS1C3* have been reported to be associated with psoriasis [48], an immune-mediated skin disease. *PSORS1C3* is located in the major histocompatability (MHC) region, and subsequent papers have found that the associations with psoriasis may be due to variants in *HLA-C* (MIM 142840) [49], [50]. Interestingly, one study has reported an epidemiologic association between psoriasis and COPD [51], and cigarette smoking is a risk factor for psoriasis as well [52]. Although there are no reports of *HLA-C* associations with COPD, alleles of other MHC class I genes, *HLA-A* and *HLA-B*, have been associated with COPD [53], [54]. The locus encompassing *PSORS1C1/3* and *HLA-C* will require additional replication studies and functional validation to confirm its role in COPD susceptibility.

Prior studies have also used eQTL analyses to identify novel genes for complex traits, including age related decline in kidney function [8] and body mass index [55]. In contrast to our study, these papers first found gene transcripts correlated with the phenotype, then tested SNPs in/near these genes for association with expression levels. We performed the cis-eQTL analysis as the initial step, then tested the eQTL SNPs for phenotype association. This limits multiple testing compared to a GWAS, enriching for eQTL SNPs which may be more likely to be associated with disease [56].

This study has several limitations. The sample size of 131 subjects, though adequate for gene expression analyses, may be underpowered to detect all potential eQTL associations. Therefore, we limited the cis-acting analysis to SNPs within 50 kb from the gene, to limit the multiple testing burden. Based on RNA sequencing data, Pickrell et al. estimate that 90% of eQTL SNPs are within 15 kb of a gene [57]. Previous papers have used a 50 kb limit to define cis-acting eQTLs [6]. Using this method, we were able to replicate published eQTL associations from other tissues and were able to identify a set of significant eQTL SNPs to carry forward for COPD association studies. However, our method would be unable to detect cis-eQTLs located >50 kb from the TSS, such as a SNP in an upstream enhancer or in the 3′ UTR of a large gene. Due to the sample size, we limited our investigation to cis-acting eQTL SNPs, as a full search for trans-acting regulatory SNPs greatly increases the number of tests performed. The literature suggests that sample sizes under 200 subjects may be inadequate to find true trans-eQTLs [58].

Several groups have compared eQTLs in different tissues from the same individual, finding both overlapping and tissue-specific eQTLs [59]–[61]. Multiple tissues are known to be important in COPD biology, including large and small airways, lung parenchyma and immune cells. By only surveying sputum, we may have missed significant eQTLs for COPD genes that are expressed in other tissues. Multiple cell types may be present in sputum, yet neutrophils have been shown to be the predominant cell type in the sputum samples from COPD subjects in ECLIPSE [16]. Despite these limitations, sputum is a clinically important tissue in COPD and is more accessible for genomic and biomarkers studies than lung tissue. Studying diseased individuals may be advantageous to identify eQTL SNPs for potential disease genes, which may only be expressed, or may be expressed at higher levels, in patients compared to healthy controls.

In conclusion, we combined genomewide SNP genotyping with genomewide expression profiling from a relevant tissue in well-characterized subjects with a common chronic disease. Using this strategy, we were able to gain insights into the functional role of SNPs previously associated through GWAS, as well as identify a potential novel disease susceptibility gene which would have been missed using standard GWAS analysis. Previous eQTL studies have provided important information about genetic control of human gene expression. Integrative genomics studies in relevant tissue from well-phenotyped individuals, as we have performed, will be required to apply this knowledge to human disease.

## Supporting Information

Figure S1 **Principal components plot of RMA expression values, demonstrating lack of batch effects based on hybridization dates or other systematic effects.**
(TIF)

Data S1 **Microarray Quality Control.**
(DOC)

Table S1 **The top cis-expression quantitative trait loci (eQTLs) in sputum samples from 131 ECLIPSE COPD subjects.** Single nucleotide polymorphism (SNP)-probe set associations with FDR-adjusted p<0.05 are shown.
(XLS)

Table S2 **Cis-expression quantitative trait locus (eQTL) single nucleotide polymorphisms (SNPs) from sputum samples from 131 ECLIPSE COPD subjects associated with COPD case-control status in combined ECLIPSE, NETT-NAS, and Norway GWAS (Cho et al. 2010).** SNPs associated at p<0.01 are shown.
(DOC)

Table S3 **Cis-expression quantitative trait locus (eQTL) single nucleotide polymorphisms (SNPs) from sputum samples from 131 ECLIPSE COPD subjects associated with COPD case-control status in a meta-analysis of ECLIPSE, NETT-NAS, and Norway GWAS (Cho et al. 2010).** SNPs with combined p<0.01 are shown.
(DOC)
