# Supplementary figures and images for: Genetics of Sputum Gene Expression in Chronic Obstructive Pulmonary Disease
Source: PLoS One. 2011 Sep 16;6(9):e24395. doi: 10.1371/journal.pone.0024395 (PMC3174957; doi:10.1371/journal.pone.0024395)

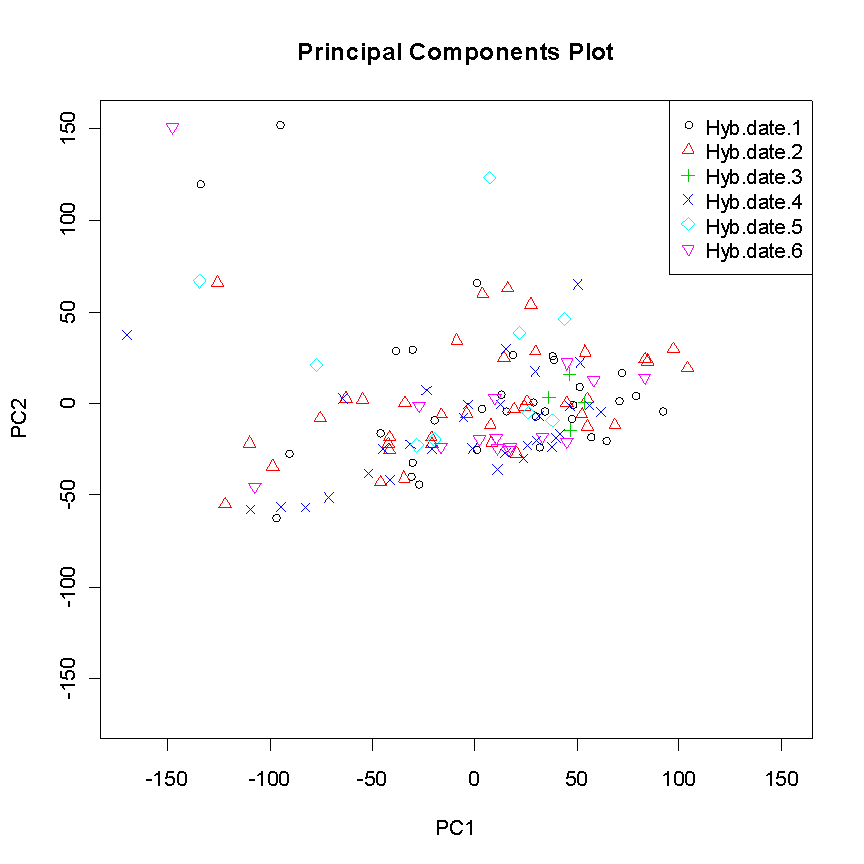

Supplement: Figure S1 — Principal components plot of RMA expression values, demonstrating lack of batch effects based on hybridization dates or other systematic effects. (TIF) [file pone.0024395.s001.tif]
