# Supplementary material for: Genetics of Sputum Gene Expression in Chronic Obstructive Pulmonary Disease
Source: PLoS One. 2011 Sep 16;6(9):e24395. doi: 10.1371/journal.pone.0024395 (PMC3174957; doi:10.1371/journal.pone.0024395)
Supplement: Data S1 — Microarray Quality Control. (DOC) [file pone.0024395.s002.doc]

Data S1. Microarray Quality Control

Quality control of microarrays was performed using the Bioconductor package affyQCReport. Correlations between RMA expression values across the 131 arrays were high (median 0.966; interquartile range 0.949, 0.977). Hoeffding’s statistic D_a_ was used for outlier detection in M-A plots. None of the 131 arrays from the eQTL exceeded the threshold of 0.015 to be considered as an outlier. Principal components analysis of RMA expression values did not reveal batch effects or other obvious patterns (Figure S1).
