# Supplementary material for: Genetics of Sputum Gene Expression in Chronic Obstructive Pulmonary Disease
Source: PLoS One. 2011 Sep 16;6(9):e24395. doi: 10.1371/journal.pone.0024395 (PMC3174957; doi:10.1371/journal.pone.0024395)
Supplement: Table S2 — Cis-expression quantitative trait locus (eQTL) single nucleotide polymorphisms (SNPs) from sputum samples from 131 ECLIPSE COPD subjects associated with COPD case-control status in combined ECLIPSE, NETT-NAS, and Norway GWAS (Cho et al. 2010). SNPs associated at p<0.01 are shown. (DOC) [file pone.0024395.s004.doc]

Supplemental Data

Qiu et al., Genetics of sputum gene expression in chronic obstructive pulmonary disease

Table S2. Cis-expression quantitative trait locus (eQTL) single nucleotide polymorphisms (SNPs) from sputum samples from 131 ECLIPSE COPD subjects associated with COPD case-control status in combined ECLIPSE, NETT-NAS, and Norway GWAS (Cho et al. 2010). SNPs associated at p<0.01 are shown.

| Chrom | BP | SNP | Affymetrix probe set | Gene | eQTL  p-value | FDR-adjusted p-value | GWAS COPD OR | GWAS COPD  p-value |
| --- | --- | --- | --- | --- | --- | --- | --- | --- |
| 1 | 40893086 | rs2744805 | 211251_x_at | *NFYC* | 0.00025 | 0.037 | 1.19 | 0.0092 |
| 1 | 46471077 | rs6692775 | 235214_at | *C1orf190* | 2.4E-06 | 0.0010 | 1.38 | 0.00040 |
| 1 | 159946268 | rs7523762 | 238452_at | *FCRLB* | 0.00011 | 0.020 | 0.80 | 0.00011 |
| 1 | 159957990 | rs1891018 | 217550_at | *ATF6* | 0.00013 | 0.023 | 0.81 | 0.0046 |
| 1 | 159980450 | rs4657094 | 217550_at | *ATF6* | 0.00037 | 0.049 | 0.76 | 0.0023 |
| 1 | 205537083 | rs1652333 | 1555950_a_at | *CD55* | 0.00012 | 0.022 | 0.85 | 0.0034 |
| 1 | 205549789 | rs7525530 | 1555950_a_at | *CD55* | 0.00019 | 0.031 | 1.21 | 0.00050 |
| 1 | 205569157 | rs6700168 | 1555950_a_at | *CD55* | 8.1E-05 | 0.016 | 0.85 | 0.0034 |
| 1 | 205569157 | rs6700168 | 201926_s_at | *CD55* | 0.00029 | 0.041 | 0.85 | 0.0034 |
| 1 | 205593216 | rs2135923 | 1555950_a_at | *CD55* | 8.1E-05 | 0.016 | 0.85 | 0.0036 |
| 1 | 205593216 | rs2135923 | 201926_s_at | *CD55* | 0.00029 | 0.041 | 0.85 | 0.0036 |
| 1 | 205603594 | rs11117564 | 1555950_a_at | *CD55* | 0.00027 | 0.039 | 1.17 | 0.0025 |
| 1 | 232597632 | rs4027040 | 225638_at | *C1orf31* | 4.5E-11 | 9.7E-08 | 1.15 | 0.0085 |
| 2 | 190229299 | rs7568449 | 217987_at | *ASNSD1* | 0.00017 | 0.028 | 0.84 | 0.0052 |
| 2 | 190254545 | rs11679767 | 217987_at | *ASNSD1* | 0.00013 | 0.023 | 0.85 | 0.0072 |
| 2 | 190351502 | rs7591929 | 227548_at | *ORMDL1* | 3.7E-20 | 7.5E-16 | 0.86 | 0.0085 |
| 2 | 190357827 | rs1899025 | 227548_at | *ORMDL1* | 3.7E-20 | 7.5E-16 | 0.85 | 0.0080 |
| 2 | 197377434 | rs2289380 | 1555439_at | *GTF3C3* | 0.00023 | 0.035 | 1.15 | 0.0088 |
| 2 | 227312149 | rs17208470 | 238933_at | *IRS1* | 4.7E-05 | 0.011 | 0.77 | 0.0022 |
| 4 | 41646007 | rs3804186 | 1562209_at | *DCAF4L1* | 3.8E-05 | 0.0092 | 1.23 | 0.0090 |
| 5 | 81630840 | rs2215128 | 227722_at | *RPS23* | 0.00028 | 0.040 | 1.16 | 0.0082 |
| 5 | 150636741 | rs17659864 | 215890_at | *GM2A* | 3.4E-07 | 0.00021 | 0.86 | 0.0096 |
| 5 | 179201385 | rs2303677 | 220341_s_at | *C5orf45* | 6.8E-05 | 0.014 | 0.84 | 0.0071 |
| 5 | 179201385 | rs2303677 | 213112_s_at | *SQSTM1* | 7.8E-05 | 0.016 | 0.84 | 0.0071 |
| 6 | 6515106 | rs1999261 | 205859_at | *LY86* | 0.00036 | 0.048 | 1.15 | 0.0080 |
| 6 | 31214156 | rs1265098 | 238997_at | *PSORS1C3* | 8.2E-05 | 0.016 | 1.18 | 0.0065 |
| 6 | 31326218 | rs3130424 | 216526_x_at | *HLA-C* | 4.9E-05 | 0.011 | 0.85 | 0.0058 |
| 6 | 125635515 | rs2318095 | 203260_at | *HDDC2* | 8.8E-07 | 0.00045 | 1.18 | 0.0056 |
| 6 | 125656120 | rs10223874 | 203260_at | *HDDC2* | 3.8E-05 | 0.0093 | 1.25 | 0.0019 |
| 6 | 125656956 | rs3734645 | 203260_at | *HDDC2* | 3.8E-05 | 0.0093 | 1.25 | 0.0016 |
| 6 | 125660257 | rs3799716 | 203260_at | *HDDC2* | 3.8E-05 | 0.0093 | 1.25 | 0.0017 |
| 6 | 125662577 | rs987168 | 203260_at | *HDDC2* | 3.7E-06 | 0.0014 | 1.27 | 0.00044 |
| 6 | 125665509 | rs12523848 | 203260_at | *HDDC2* | 1.7E-06 | 0.00075 | 1.19 | 0.0033 |
| 7 | 99423377 | rs1076237 | 214670_at | *ZKSCAN1* | 0.00020 | 0.031 | 1.17 | 0.0051 |
| 7 | 99433358 | rs10267212 | 214670_at | *ZKSCAN1* | 0.00018 | 0.029 | 1.16 | 0.0081 |
| 7 | 99494863 | rs12705070 | 214670_at | *ZKSCAN1* | 0.00025 | 0.038 | 1.17 | 0.0061 |
| 10 | 12954527 | rs4750277 | 1559990_at | *LOC283070* | 7.9E-06 | 0.0026 | 0.83 | 0.0097 |
| 11 | 129198876 | rs11608143 | 230323_s_at | *TMEM45B* | 2.2E-05 | 0.0060 | 1.23 | 0.0081 |
| 11 | 129198876 | rs11608143 | 226226_at | *TMEM45B* | 0.00034 | 0.046 | 1.23 | 0.0081 |
| 11 | 129204066 | rs10894147 | 230323_s_at | *TMEM45B* | 2.2E-05 | 0.0060 | 1.23 | 0.0085 |
| 11 | 129204066 | rs10894147 | 226226_at | *TMEM45B* | 0.00034 | 0.046 | 1.23 | 0.0085 |
| 12 | 21469826 | rs12817513 | 231173_at | *PYROXD1* | 0.00023 | 0.035 | 1.15 | 0.0095 |
| 12 | 21474509 | rs10437776 | 231173_at | *PYROXD1* | 0.00020 | 0.032 | 1.15 | 0.0079 |
| 12 | 21507490 | rs12424924 | 231173_at | *PYROXD1* | 0.00022 | 0.035 | 1.19 | 0.0038 |
| 12 | 21520058 | rs3752648 | 231173_at | *PYROXD1* | 6.9E-08 | 5.5E-05 | 1.15 | 0.0066 |
| 14 | 62896438 | rs10143004 | 203338_at | *PPP2R5E* | 8.1E-05 | 0.016 | 0.77 | 0.0065 |
| 14 | 62899422 | rs12323683 | 203338_at | *PPP2R5E* | 8.1E-05 | 0.016 | 0.77 | 0.0077 |
| 14 | 62901927 | rs12435569 | 203338_at | *PPP2R5E* | 0.00032 | 0.045 | 0.78 | 0.0087 |
| 14 | 62903730 | rs10140256 | 203338_at | *PPP2R5E* | 0.00032 | 0.044 | 0.77 | 0.0072 |
| 14 | 62921930 | rs8004556 | 203338_at | *PPP2R5E* | 0.00032 | 0.044 | 0.78 | 0.0076 |
| 14 | 62923992 | rs10135846 | 203338_at | *PPP2R5E* | 0.00032 | 0.044 | 0.72 | 0.0065 |
| 15 | 41726934 | rs8042868 | 1561405_s_at | *CATSPER2* | 3.3E-06 | 0.0013 | 0.71 | 0.00018 |
| 15 | 41726934 | rs8042868 | 1553323_a_at | *CATSPER2* | 3.1E-05 | 0.0078 | 0.71 | 0.00018 |
| 15 | 76532762 | rs2656069 | 1555476_at | *IREB2* | 0.00030 | 0.042 | 0.75 | 6.8E-06 |
| 15 | 76681394 | rs1051730 | 206533_at | *CHRNA5* | 0.00015 | 0.026 | 1.29 | 2.8E-06 |
| 15 | 99662447 | rs2073595 | 242146_at | *SNRPA1* | 4.4E-05 | 0.010 | 0.81 | 0.0018 |
| 15 | 99675070 | rs7169313 | 242146_at | *SNRPA1* | 4.5E-05 | 0.010 | 0.82 | 0.0069 |
| 17 | 5225494 | rs1065483 | 230274_s_at | *NUP88* | 3.9E-13 | 1.3E-09 | 1.17 | 0.0030 |
| 17 | 5260344 | rs2301740 | 230274_s_at | *NUP88* | 9.1E-14 | 3.5E-10 | 1.17 | 0.0035 |
| 18 | 2528545 | rs2347279 | 219698_s_at | *METTL4* | 0.00013 | 0.024 | 0.83 | 0.0025 |
| 18 | 2560155 | rs1878553 | 219698_s_at | *METTL4* | 8.7E-05 | 0.017 | 0.83 | 0.0028 |
| 18 | 42200835 | rs16978548 | 230143_at | *RNF165* | 0.00012 | 0.022 | 0.81 | 0.0049 |
| 19 | 18568973 | rs7250623 | 218958_at | *C19orf60* | 2.2E-05 | 0.0059 | 0.87 | 0.0072 |
| 19 | 46777713 | rs2302188 | 214907_at | *CEACAM21* | 0.00016 | 0.027 | 0.85 | 0.0075 |
