# Supplementary material for: Genetics of Sputum Gene Expression in Chronic Obstructive Pulmonary Disease
Source: PLoS One. 2011 Sep 16;6(9):e24395. doi: 10.1371/journal.pone.0024395 (PMC3174957; doi:10.1371/journal.pone.0024395)
Supplement: Table S3 — Cis-expression quantitative trait locus (eQTL) single nucleotide polymorphisms (SNPs) from sputum samples from 131 ECLIPSE COPD subjects associated with COPD case-control status in a meta-analysis of ECLIPSE, NETT-NAS, and Norway GWAS (Cho et al. 2010). SNPs with combined p<0.01 are shown. (DOC) [file pone.0024395.s005.doc]

Table S3. Cis-expression quantitative trait locus (eQTL) single nucleotide polymorphisms (SNPs) from sputum samples from 131 ECLIPSE COPD subjects associated with COPD case-control status in a meta-analysis of ECLIPSE, NETT-NAS, and Norway GWAS (Cho et al. 2010). SNPs with combined p<0.01 are shown.

| Chrom | SNP | Affymetrix probe set | Gene | eQTL  p-value | FDR-adjusted p-value | ECLIPSE COPD OR | ECLIPSE COPD  p-value | NETT-NAS COPD OR | NETT-NAS COPD  p-value | Norway COPD OR | Norway COPD  p-value | COPD GWAS meta-analysis  p-value |
| --- | --- | --- | --- | --- | --- | --- | --- | --- | --- | --- | --- | --- |
| 1 | rs11117564 | 1555950_a_at | *CD55* | 0.00027 | 0.039 | 1.06 | 0.63 | 1.39 | 0.0080 | 1.20 | 0.032 | 0.0019 |
| 1 | rs1652333 | 1555950_a_at | *CD55* | 0.00012 | 0.022 | 0.84 | 0.19 | 0.78 | 0.061 | 0.85 | 0.076 | 0.0042 |
| 1 | rs2135923 | 1555950_a_at | *CD55* | 8.1E-05 | 0.016 | 0.86 | 0.25 | 0.76 | 0.046 | 0.85 | 0.068 | 0.0039 |
| 1 | rs2135923 | 201926_s_at | *CD55* | 0.00029 | 0.041 | 0.86 | 0.25 | 0.76 | 0.046 | 0.85 | 0.068 | 0.0039 |
| 1 | rs4027040 | 225638_at | *C1orf31* | 4.5E-11 | 9.7E-08 | 1.19 | 0.16 | 1.16 | 0.23 | 1.22 | 0.018 | 0.0030 |
| 1 | rs6429566 | 1559003_a_at | *LOC126661* | 3.4E-05 | 0.0084 | 0.83 | 0.14 | 0.75 | 0.046 | 0.89 | 0.19 | 0.0079 |
| 1 | rs6429566 | 236900_x_at | *LOC126661* | 0.00037 | 0.049 | 0.83 | 0.14 | 0.75 | 0.046 | 0.89 | 0.19 | 0.0079 |
| 1 | rs6692775 | 235214_at | *C1orf190* | 2.4E-06 | 0.0010 | 1.21 | 0.21 | 1.59 | 0.0022 | NA | NA | 0.0019 |
| 1 | rs6700168 | 1555950_a_at | *CD55* | 8.1E-05 | 0.016 | 0.86 | 0.25 | 0.77 | 0.046 | 0.85 | 0.068 | 0.0039 |
| 1 | rs6700168 | 201926_s_at | *CD55* | 0.00029 | 0.041 | 0.86 | 0.25 | 0.77 | 0.046 | 0.85 | 0.068 | 0.0039 |
| 1 | rs7523762 | 238452_at | *FCRLB* | 0.00011 | 0.020 | 0.75 | 0.034 | 0.88 | 0.35 | 0.77 | 0.0046 | 0.00042 |
| 1 | rs7525530 | 1555950_a_at | *CD55* | 0.00019 | 0.031 | 1.13 | 0.35 | 1.32 | 0.025 | 1.23 | 0.014 | 0.00081 |
| 2 | rs11679767 | 217987_at | *ASNSD1* | 0.00013 | 0.023 | 0.85 | 0.25 | 0.80 | 0.11 | 0.84 | 0.064 | 0.0069 |
| 2 | rs17208470 | 238933_at | *IRS1* | 4.7E-05 | 0.011 | 0.78 | 0.20 | 0.71 | 0.095 | 0.70 | 0.0093 | 0.00091 |
| 2 | rs1899025 | 227548_at | *ORMDL1* | 3.7E-20 | 7.5E-16 | 0.83 | 0.19 | 0.78 | 0.075 | 0.84 | 0.066 | 0.0042 |
| 2 | rs7568449 | 217987_at | *ASNSD1* | 0.00017 | 0.028 | 0.82 | 0.16 | 0.90 | 0.47 | 0.79 | 0.015 | 0.0050 |
| 2 | rs7591929 | 227548_at | *ORMDL1* | 3.7E-20 | 7.5E-16 | 0.83 | 0.19 | 0.78 | 0.077 | 0.84 | 0.066 | 0.0043 |
| 4 | rs3804186 | 1562209_at | *DCAF4L1* | 3.8E-05 | 0.0092 | 1.22 | 0.31 | 1.08 | 0.69 | 1.42 | 0.0049 | 0.0072 |
| 5 | rs1469684 | 215890_at | *GM2A* | 3.6E-07 | 0.00022 | 0.70 | 0.0080 | 0.78 | 0.079 | NA | NA | 0.0019 |
| 5 | rs17659864 | 215890_at | *GM2A* | 3.4E-07 | 0.00021 | 0.70 | 0.0080 | 0.78 | 0.088 | 0.85 | 0.084 | 0.00057 |
| 5 | rs2075783 | 215890_at | *GM2A* | 3.5E-15 | 1.9E-11 | 0.80 | 0.10 | 0.90 | 0.43 | 0.83 | 0.040 | 0.0073 |
| 5 | rs226205 | 227722_at | *RPS23* | 3.7E-10 | 5.9E-07 | 0.77 | 0.063 | 0.78 | 0.088 | 0.83 | 0.051 | 0.0015 |
| 5 | rs226206 | 227722_at | *RPS23* | 3.1E-11 | 7.0E-08 | 0.79 | 0.067 | 0.76 | 0.037 | 0.87 | 0.11 | 0.0019 |
| 5 | rs246498 | 215891_s_at | *GM2A* | 0.00024 | 0.037 | 1.15 | 0.27 | 1.29 | 0.038 | 1.17 | 0.059 | 0.0032 |
| 5 | rs246498 | 33646_g_at | *GM2A* | 0.00029 | 0.042 | 1.15 | 0.27 | 1.29 | 0.038 | 1.17 | 0.059 | 0.0032 |
| 5 | rs749401 | 227722_at | *RPS23* | 1.0E-08 | 1.0E-05 | 0.80 | 0.10 | 0.78 | 0.077 | 0.88 | 0.19 | 0.0083 |
| 6 | rs12523848 | 203260_at | *HDDC2* | 1.7E-06 | 0.00075 | 0.95 | 0.70 | 1.14 | 0.33 | 1.38 | 0.00061 | 0.0082 |
| 6 | rs1999261 | 205859_at | *LY86* | 0.00036 | 0.048 | 1.24 | 0.096 | 1.21 | 0.11 | 1.31 | 0.0026 | 0.00018 |
| 6 | rs2206926 | 244407_at | *CYP39A1* | 7.1E-06 | 0.0024 | 1.21 | 0.40 | 1.68 | 0.011 | 1.24 | 0.20 | 0.0077 |
| 6 | rs2318095 | 203260_at | *HDDC2* | 8.8E-07 | 0.00045 | 0.97 | 0.83 | 1.17 | 0.25 | 1.34 | 0.0019 | 0.0088 |
| 6 | rs987168 | 203260_at | *HDDC2* | 3.7E-06 | 0.0014 | 0.99 | 0.93 | 1.07 | 0.69 | 1.47 | 0.00030 | 0.0081 |
| 8 | rs3758041 | 1557582_at | *BIN3* | 1.7E-08 | 1.6E-05 | 0.84 | 0.14 | 0.93 | 0.57 | 0.81 | 0.015 | 0.0062 |
| 9 | rs3752955 | 219147_s_at | *C9orf95* | 8.2E-10 | 1.2E-06 | 1.25 | 0.062 | 1.20 | 0.12 | 0.85 | 0.045 | 0.0016 |
| 9 | rs7022554 | 242961_x_at | *DDX58* | 6.6E-09 | 7.2E-06 | 0.77 | 0.034 | NA | NA | 0.87 | 0.10 | 0.0099 |
| 10 | rs7895270 | 213369_at | *PCDH21* | 0.00025 | 0.037 | 0.72 | 0.016 | 0.95 | 0.74 | 0.83 | 0.049 | 0.0053 |
| 11 | rs2078786 | 204981_at | *SLC22A18* | 0.00023 | 0.035 | 0.92 | 0.50 | 0.88 | 0.31 | 0.79 | 0.0064 | 0.0058 |
| 12 | rs1025607 | 229596_at | *AMDHD1* | 8.2E-05 | 0.016 | 1.03 | 0.85 | 1.13 | 0.34 | 1.29 | 0.0036 | 0.0089 |
| 14 | rs10135846 | 203338_at | *PPP2R5E* | 0.00032 | 0.044 | 0.57 | 0.0043 | NA | NA | 0.85 | 0.27 | 0.0095 |
| 14 | rs10140256 | 203338_at | *PPP2R5E* | 0.00032 | 0.044 | 0.57 | 0.0044 | 0.73 | 0.19 | 0.88 | 0.39 | 0.0070 |
| 14 | rs10143004 | 203338_at | *PPP2R5E* | 8.1E-05 | 0.016 | 0.58 | 0.0058 | 0.77 | 0.27 | 0.83 | 0.22 | 0.0043 |
| 14 | rs12323683 | 203338_at | *PPP2R5E* | 8.1E-05 | 0.016 | 0.59 | 0.0092 | 0.72 | 0.16 | 0.85 | 0.31 | 0.0060 |
| 14 | rs12435569 | 203338_at | *PPP2R5E* | 0.00032 | 0.045 | 0.60 | 0.0098 | 0.75 | 0.21 | 0.82 | 0.18 | 0.0037 |
| 14 | rs8004556 | 203338_at | *PPP2R5E* | 0.00032 | 0.044 | 0.57 | 0.0043 | 0.76 | 0.25 | 0.85 | 0.27 | 0.0046 |
| 15 | rs1051730 | 206533_at | *CHRNA5* | 0.00015 | 0.026 | 1.03 | 0.84 | 1.34 | 0.021 | 1.35 | 0.00043 | 0.00022 |
| 15 | rs2656069 | 1555476_at | *IREB2* | 0.00030 | 0.042 | 0.68 | 0.010 | 0.64 | 0.0026 | 0.86 | 0.16 | 0.00013 |
| 15 | rs8042868 | 1561405_s_at | *CATSPER2* | 3.3E-06 | 0.0013 | 0.98 | 0.92 | 0.60 | 0.010 | 0.66 | 0.012 | 0.0018 |
| 15 | rs8042868 | 1553323_a_at | *CATSPER2* | 3.1E-05 | 0.0078 | 0.98 | 0.92 | 0.60 | 0.010 | 0.66 | 0.012 | 0.0018 |
| 16 | rs899729 | 203028_s_at | *CYBA* | 0.00038 | 0.050 | 0.98 | 0.90 | 0.78 | 0.058 | 0.82 | 0.022 | 0.0087 |
| 17 | rs1065483 | 230274_s_at | *NUP88* | 3.9E-13 | 1.3E-09 | 1.01 | 0.90 | 1.36 | 0.013 | 1.16 | 0.071 | 0.0093 |
| 17 | rs9907506 | 206969_at | *KRT34* | 0.00037 | 0.049 | 1.11 | 0.43 | 1.42 | 0.011 | 1.16 | 0.10 | 0.0045 |
| 18 | rs1878553 | 219698_s_at | *METTL4* | 8.7E-05 | 0.017 | 0.78 | 0.069 | 0.93 | 0.62 | 0.73 | 0.0026 | 0.0012 |
| 18 | rs2347279 | 219698_s_at | *METTL4* | 0.00013 | 0.024 | 0.77 | 0.066 | 0.97 | 0.81 | 0.71 | 0.00088 | 0.00090 |
| 19 | rs2302188 | 214907_at | *CEACAM21* | 0.00016 | 0.027 | 0.87 | 0.30 | 0.74 | 0.036 | 0.82 | 0.049 | 0.0029 |
| 19 | rs4803481 | 214907_at | *CEACAM21* | 0.00012 | 0.023 | 0.86 | 0.28 | 0.79 | 0.11 | 0.81 | 0.035 | 0.0043 |
| 19 | rs4803481 | 216605_s_at | *CEACAM21* | 0.00030 | 0.043 | 0.86 | 0.28 | 0.79 | 0.11 | 0.81 | 0.035 | 0.0043 |
| 22 | rs6001881 | 226802_s_at | *LOC96610* | 0.00029 | 0.041 | 0.88 | 0.42 | 0.73 | 0.049 | 0.79 | 0.045 | 0.0048 |
